# Author Correction: Mapping effective connectivity of human amygdala subdivisions with intracranial stimulation

**DOI:** 10.1038/s41467-022-33286-w

**Published:** 2022-09-27

**Authors:** Masahiro Sawada, Ralph Adolphs, Brian J. Dlouhy, Rick L. Jenison, Ariane E. Rhone, Christopher K. Kovach, Jeremy, D. W. Greenlee, Matthew A. Howard III, Hiroyuki Oya

**Affiliations:** 1 Department of Neurosurgery, Carver College of Medicine, University of Iowa, Iowa City, IA USA; 2 Department of Neurosurgery, Tazuke Kofukai Medical Research Institute and Kitano Hospital, Osaka, Japan; 3 Division of Humanities and Social Sciences, California Institute of Technology, Pasadena, CA USA; 4 Iowa Neuroscience Institute, University of Iowa, Iowa City, IA USA; 5 Department of Neuroscience, University of Wisconsin - Madison, Madison, WI USA; 6 Pappajohn Biomedical Institute, University of Iowa, Iowa City, IA USA

**Keywords:** Depression, Epilepsy, Amygdala, Human behaviour

Correction to: *Nature Communications* 10.1038/s41467-022-32644-y, published online 20 August 2022

The original version of the Supplementary Information associated with the Article omitted Supplementary Tables 1–3. The HTML has been updated to include a corrected version of the Supplementary Information.

## Supplementary information

Updated Supplementary Information

