# Supplementary material for: Mapping effective connectivity of human amygdala subdivisions with intracranial stimulation
Source: Nat Commun. 2022 Aug 20;13:4909. doi: 10.1038/s41467-022-32644-y (PMC9392722; doi:10.1038/s41467-022-32644-y)
Supplement: Supplementary file 1 — Supplementary Information [file 41467_2022_32644_MOESM1_ESM.pdf]

Supplemantary information

ROI averaged es-TT responses

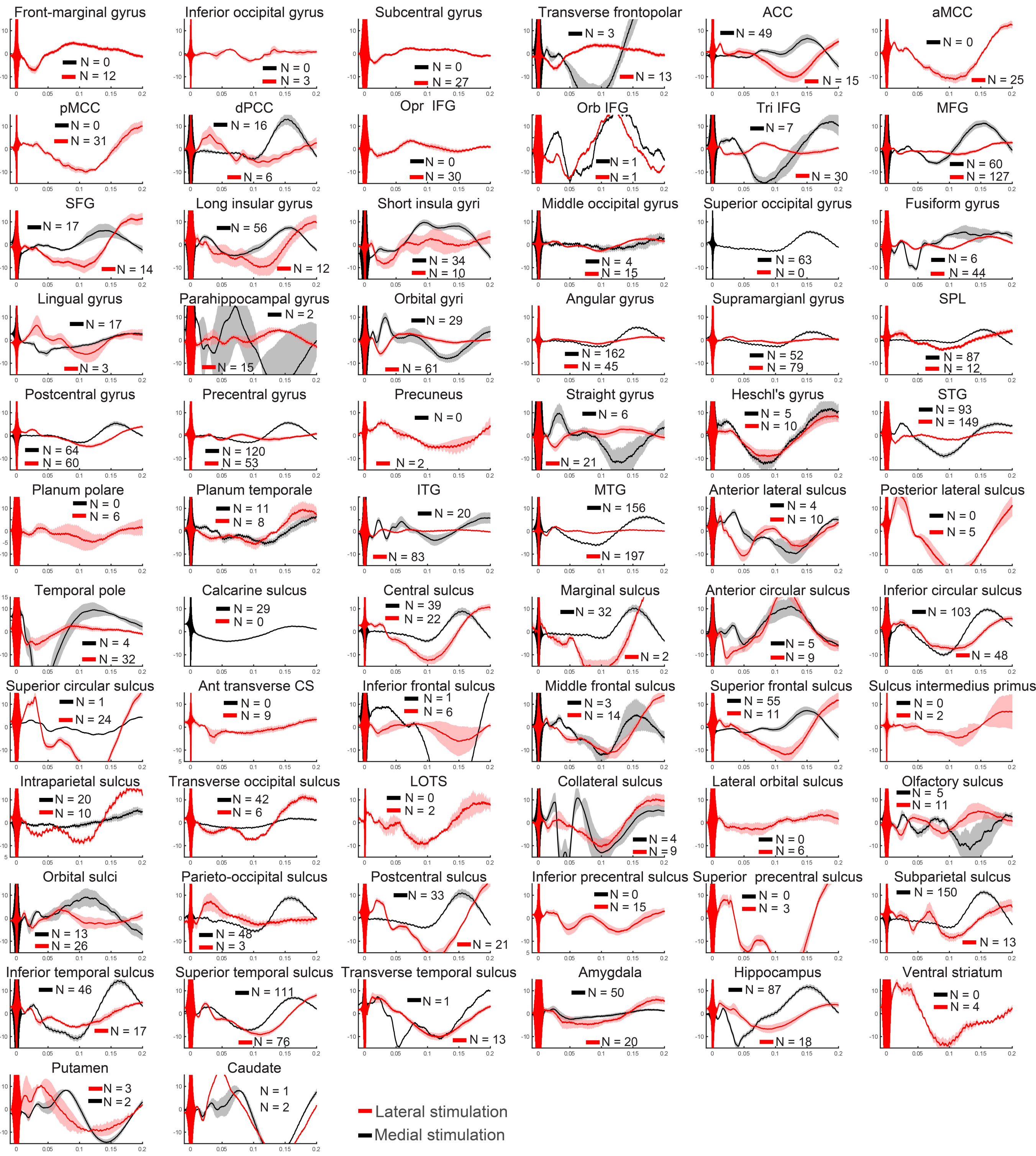

Supplementary Figure 1 | Overall ROI averaged intracranial EPs waveform from all patients. Red lines and shadings: lateral amygdala stimulation. Gray lines and shadings: medial amygdala stimulation. Number of recording channels within each ROIs are also shown in each panel (N). x-axis indicates time in s. y-axis indicates normalized magnitude in SD unit. Thick lines indicate mean, and shading area indicates SE. x- and y-axis ranges are same for all panels. For abbreviations (from Destrieux, 2010); Opr IFG: Opercular part of inferior frontal gyrus, Orb IFG: Orbital part of inferior frontal gyrus, Tri IFG: Triangular part of inferior frontal gyrus, SPL: superior parietal lobule, Ant transverse CS: Anterior transverse collateral sulcus, LOTS: lateral occipito-temporal sulcus.

Amygdala stimulation es-fMRI response
(Variable shape regression)

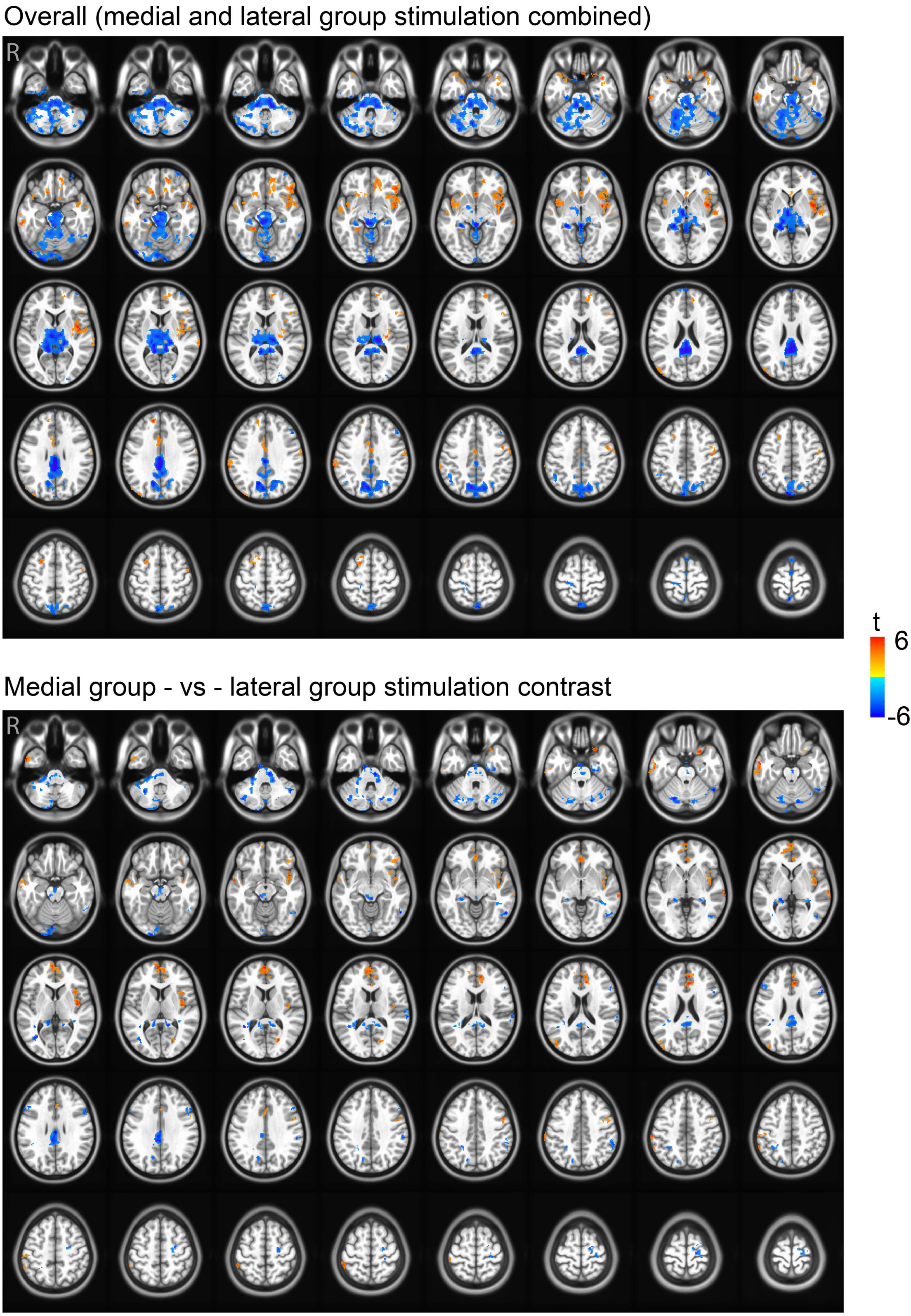

Supplementary Figure 2 | Overall es-fMRI response. Results from mixed-effect group analysis is shown in the MNI template brain volume. Upper panel shows overall es-fMRI response (all stimulation sites were combined). Lower panel shows statistical maps from medial group versus lateral group amygdala stimulation contrast. Cluster-size thresholding were applied (3dClustSim, two-sided). Statistical maps were thresholded at cluster size alpha = 0.02 with primary voxel-wise threshold at P = 0.01. For the creation of brain backgrounds, we used ICBM152 Nonlinear template brain (obtained from http://nist.mni.mcgill.ca/icbm-152-nonlinear-atlases-2009/) under Copyright (C) 1993–2004 Louis Collins, McConnell Brain Imaging Centre, Montreal Neurological Institute, McGill University.

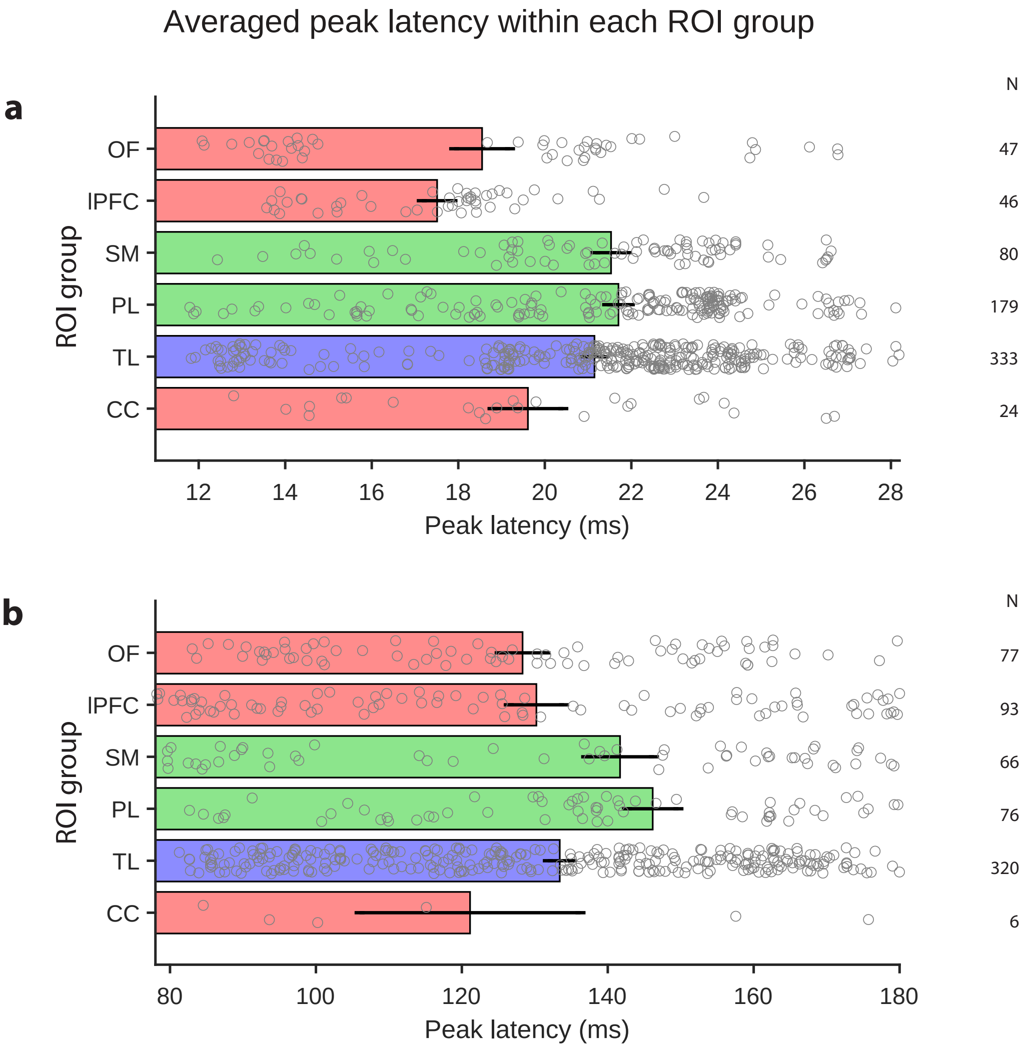

Supplementary Figure 3 | Latency distribution of Evoked potentials (EPs). ROI abbreviations are the same as in main Fig. 3. **(a)** Peak latencies of N/P15 from medial amygdala stimulation. One-way ANOVA showed significant difference in latencies among the ROI groups, F(5, 703) = 14.6, P = 1.34 x 10^{-13}. Mean and SE (bars and errorbars) are shown together with each data points represented by gray circles. Tukey's tests indicate significant difference between OF-SM (P = 1.66 x 10^{-4}), OF-PL (P = 2.91 x 10^{-6}), OF-TL (P = 9.47 x 10^{-5}), lPLF-SM (P = 6.09 x 10^{-8}), lPFC-PL (P = 9.02 x 10^{-11}) and lPFC-TL (P = 5.68 x 10^{-9}). **(b)** Peak latencies of P150 from lateral amygdala stimulation. For (a) and (b), mean and SE are shown. One-way ANOVA showed significant difference in latencies among the ROI groups, F(5, 632) = 4.02, P = 0.0013. Tukey's tests indicate significant difference between OF-PL (P = 0.006), LPFC-PL (P = 0.013) and PL-TL (P = 0.019). (OF + lPFC + CC) -vs - (SM + PL + TL) contrast also showed significant difference (two-sided t-test, P = 0.0067). N indicates number of used recording channels in the analyses. Number of EPs (N) are also presented on the right.

es-TT latency distribution (ACC and dPCC)

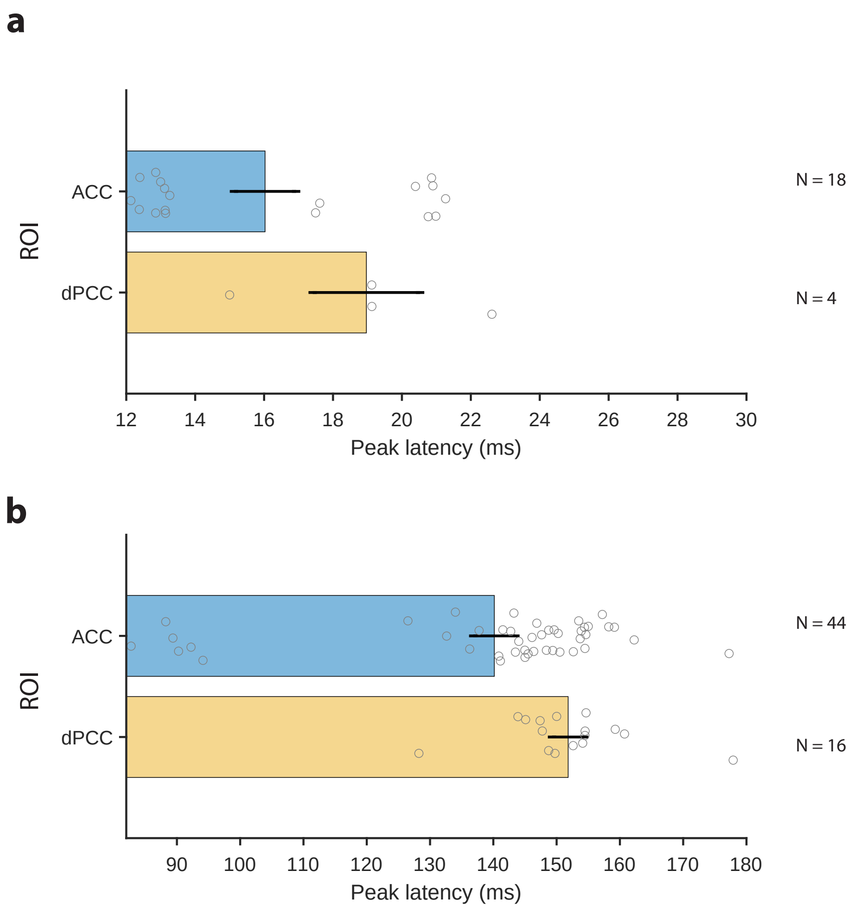

Supplementary Figure 4 | Latency difference between ACC and dPCC. (a) Average peak latency and its SE of N/P15 component of evoked potentials in the anterior cingulate cortex(ACC) and dorsal posterior cingulate cortex (dPCC) for lateral amygdala stimulation. Indivisdual data points were represented by gray circles. N indicates the number of used EPs in the analyses. **(b)** Same as (a) for P150 component. Medial amygdala stimulation. Mean and SE are shown as bars and errorbars.

Negative partial correlation network

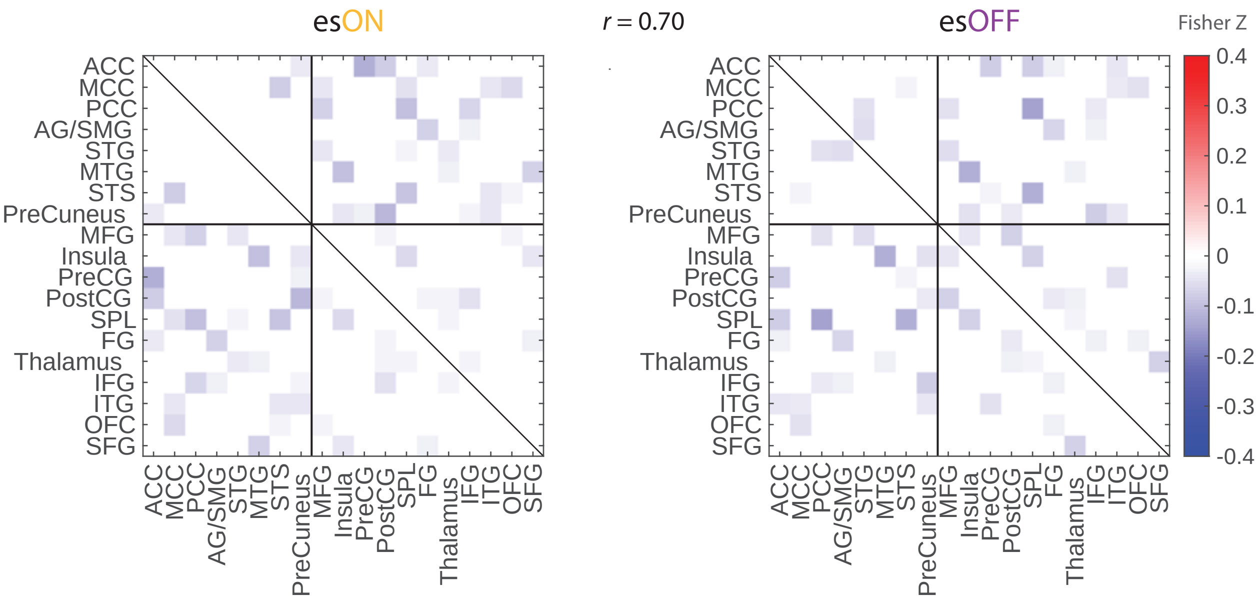

Supplementary Figure 5 | es-fMRI negative partial correlation network. BOLD time series negative partial correlation network in electrical stimulation on (esON) and off (esOFF) periods are shown. Parcellation is found by Leiden community detection algorithm (see Methods). r-values indicates Pearson correlation coefficient between esON and esOFF connectivity matrix. Color encodes Fisher-Z score of partial correlation coefficients. Thresholded at FDR = 0.05.

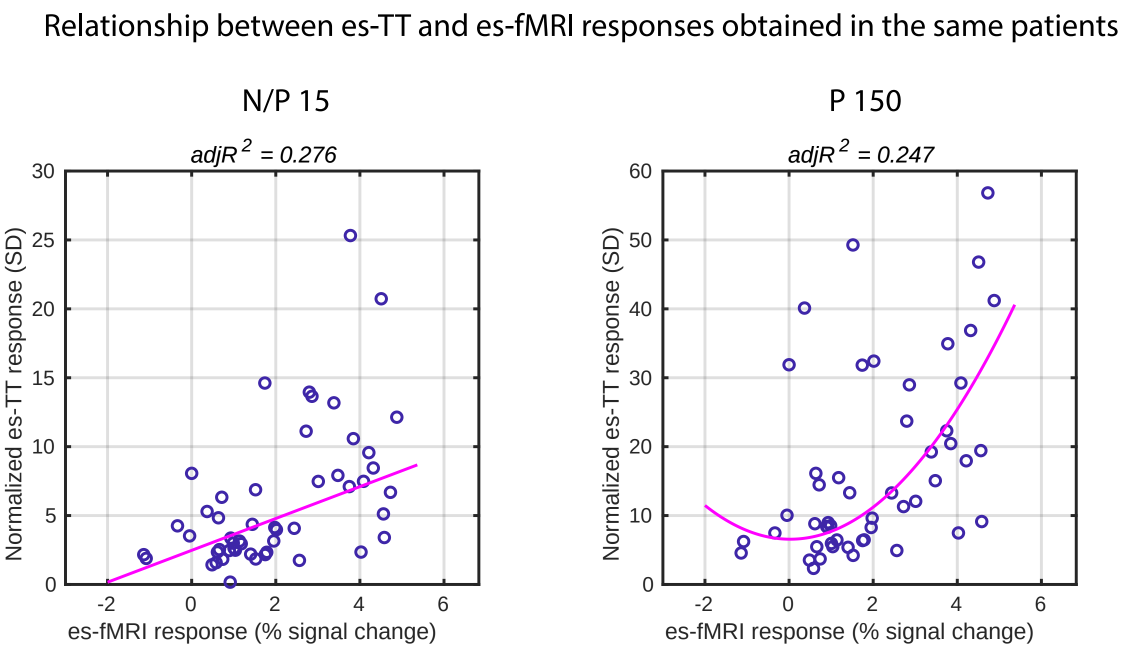

Supplementary Figure 6 | Correlation between es-TT and es-fMRI response. Correlation between electrophysiological (es-TT) and BOLD (es-fMRI) responses obtained in the same patients (N = 2). Each dot represents mean response in a ROI where both recordings (es-TT asnd es-fMRI) were available. Right and left scatter-plots are for P150 and N/P15, respectively. Adjusted R squares were presented on top of axis box. Asterisks indicates significant fit (95 % confidence bound). N/P15 used linear fit due to insignificant coefficient for quadratic component.

Conditional Granger Causality analyses
(Lateral group stimulation)

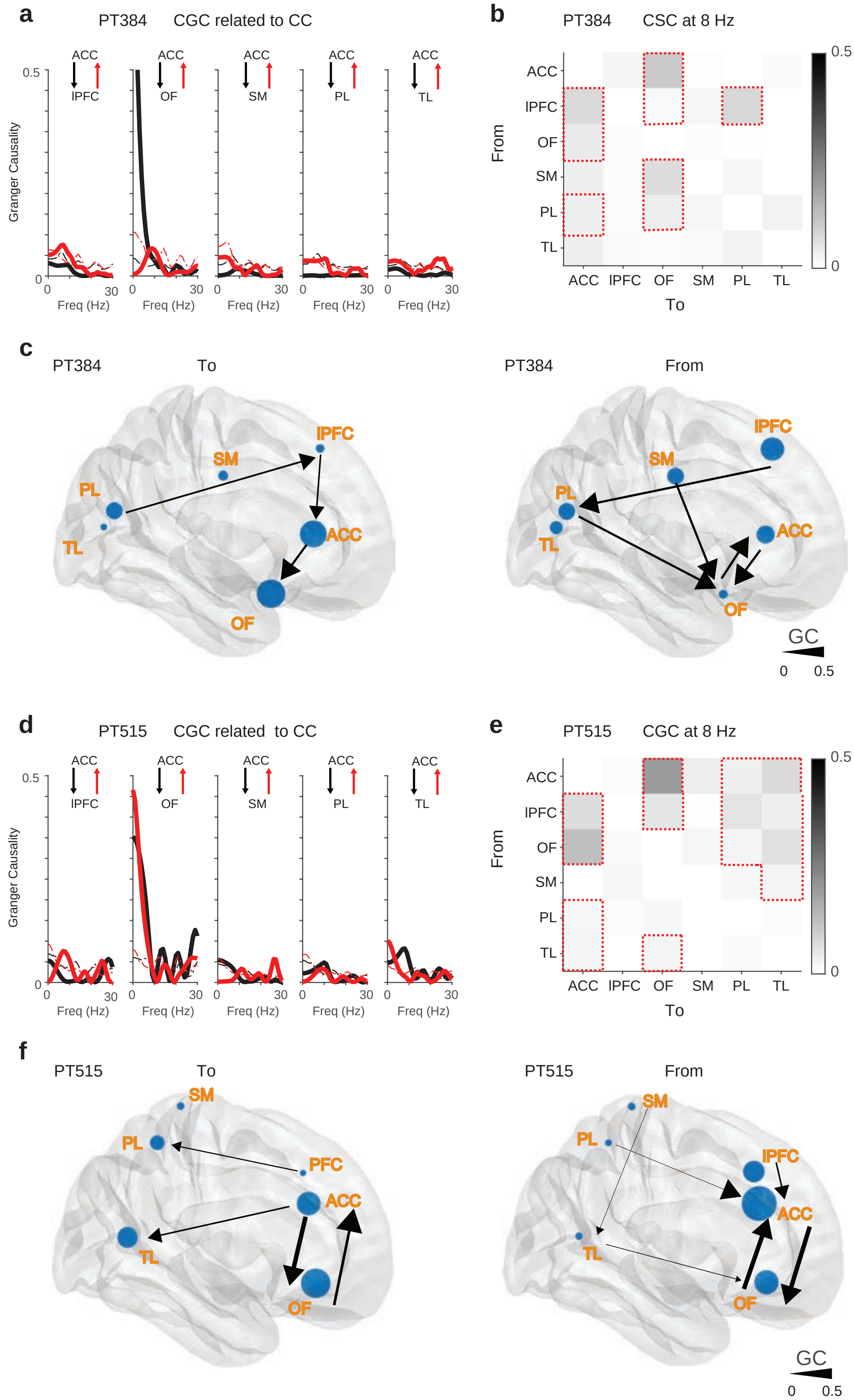

Supplementary Figure 7 | Results of Conditional Granger Causality (CGC) of intracranial field potentials in two patients for lateral group amygdala stimulation. (a) Mean spectral conditional Granger causality related to anterior cingulate cortex (CC) in PT384. x-axis indicates iEEG frequency (Hz) and y-axis indicates magnitude of the causality measurements. Solid lines represent CGC calculated from actual data and dotted lines represent 95 % CGC values found with phase-randomized surrogate data. **(b)** CGC among 5 ROI groups. Peak CGC values at 8 Hz were shown. Colorbar represents CGC values at 8 Hz. The cells in the matrix surrounded by the red dotted lines indicates significant CGC. **(c)** Effective connectivity (CGC) plotted on the MNI brain among ROIs. Thickness of the arrows are proportional to the CGC values of the connections, and size of the circles represent sum of the inflow (right panel) to and outflow (left panel) from the ROIs (In-degrees and Out-degrees, respectively). **(d)**, **(e)** and **(f)** Same as (a) (b) and (c) above but for PT515. For the creation of brain backgrounds in panel (c) and (f), we used Conte69.32k surface mesh (obtained from https://biomedia.doc.ic.ac.uk/brain-parcellationsurvey/) under WU-Minn HCP Consortium Open Access Data Use Terms.

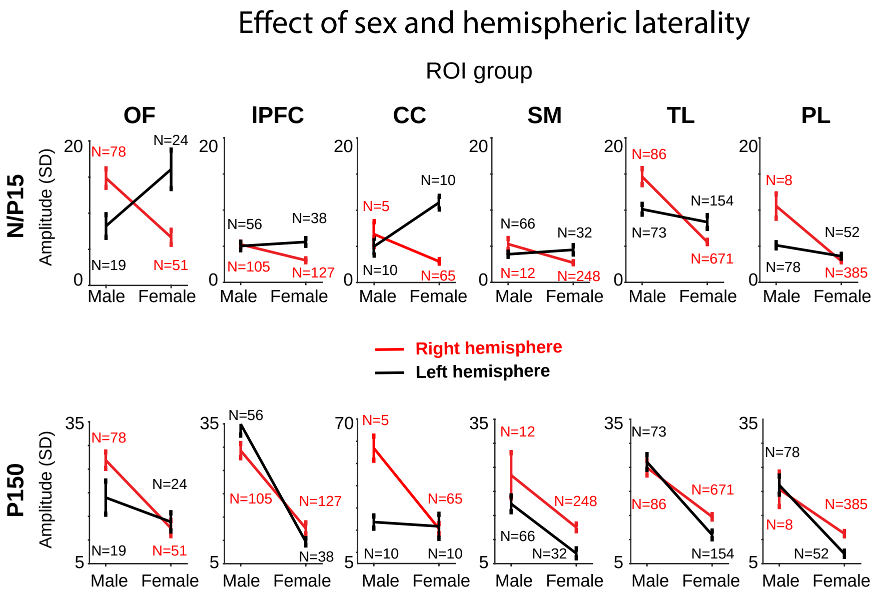

	Hemi	Gender	ROI group						
			OF	IPFC	CC	SM	TL	PL	Mean
N/P15	Rt	Male	14.7 (1.4)	5.2 (0.3)	6.7 (1.7)	5.3 (0.8)	14.6 (1.1)	10.6 (1.7)	9.5 (1.2)
		Female	6.7 (0.9)	3.1 (0.1)	2.9 (0.2)	2.7 (0.3)	5.6 (0.2)	2.9 (0.1)	4 (0.3)
	Lt	Male	8.3 (1.6)	5.0 (0.4)	7.3 (1.8)	4.9 (0.5)	10.3 (0.7)	5.1 (0.4)	6.8 (0.9)
		Female	16.0 (2.7)	5.7 (0.4)	7.6 (1.6)	4.5 (0.5)	8.4 (0.9)	3.6 (0.3)	7.6 (1.1)
		Mean	11.4 (1.7)	4.8 (0.3)	6.1 (1.3)	4.4 (0.5)	9.7 (0.7)	5.6 (0.6)	7.0 (0.9)
P150	Rt	Male	27.9 (1.7)	29.3 (1.4)	56.7 (5.4)	23.4 (4.9)	24.9 (1.5)	20.4 (3.7)	30.4 (3.1)
		Female	12.5 (1.6)	12.6 (1.1)	20.6 (1.9)	12.5 (0.8)	14.8 (0.5)	11.3 (0.8)	14.1 (1.1)
	Lt	Male	19.0 (3.5)	35.0 (2.3)	20.5 (2.8)	17.4 (1.6)	26.1 (1.5)	21.3 (1.9)	23.2 (2.3)
		Female	13.8 (1.9)	9.7 (0.8)	15.6 (4.3)	7.2 (0.9)	11.0 (0.9)	7.1 (0.6)	10.7 (1.6)
		Mean	18.3 (2.2)	21.7 (1.4)	28.4 (3.6)	15.1 (2.1)	19.2 (1.1)	15.0 (1.8)	19.6 (2.0)

Supplementary Figure 8 | Effect of Sex and Hemispheric laterality.
es-TT response magnitudes (mean+SE) showing effect sizes of two factors (Sex and Hemispheric laterality) for each ROI group. X-axis: Sex, Y-axis: mean es-TT EPs response amplitude in SD unit. N = Number of evoked potentials. Medial and lateral group amygdala stimulations were combined. Only effect sizes are reported. Table below shows mean and SE values for gender and hemispheric laterality separately within each ROI group. Note that only EPs that were obtained in the hemisphere ipsilateral to stimulation were considered (see Methods).

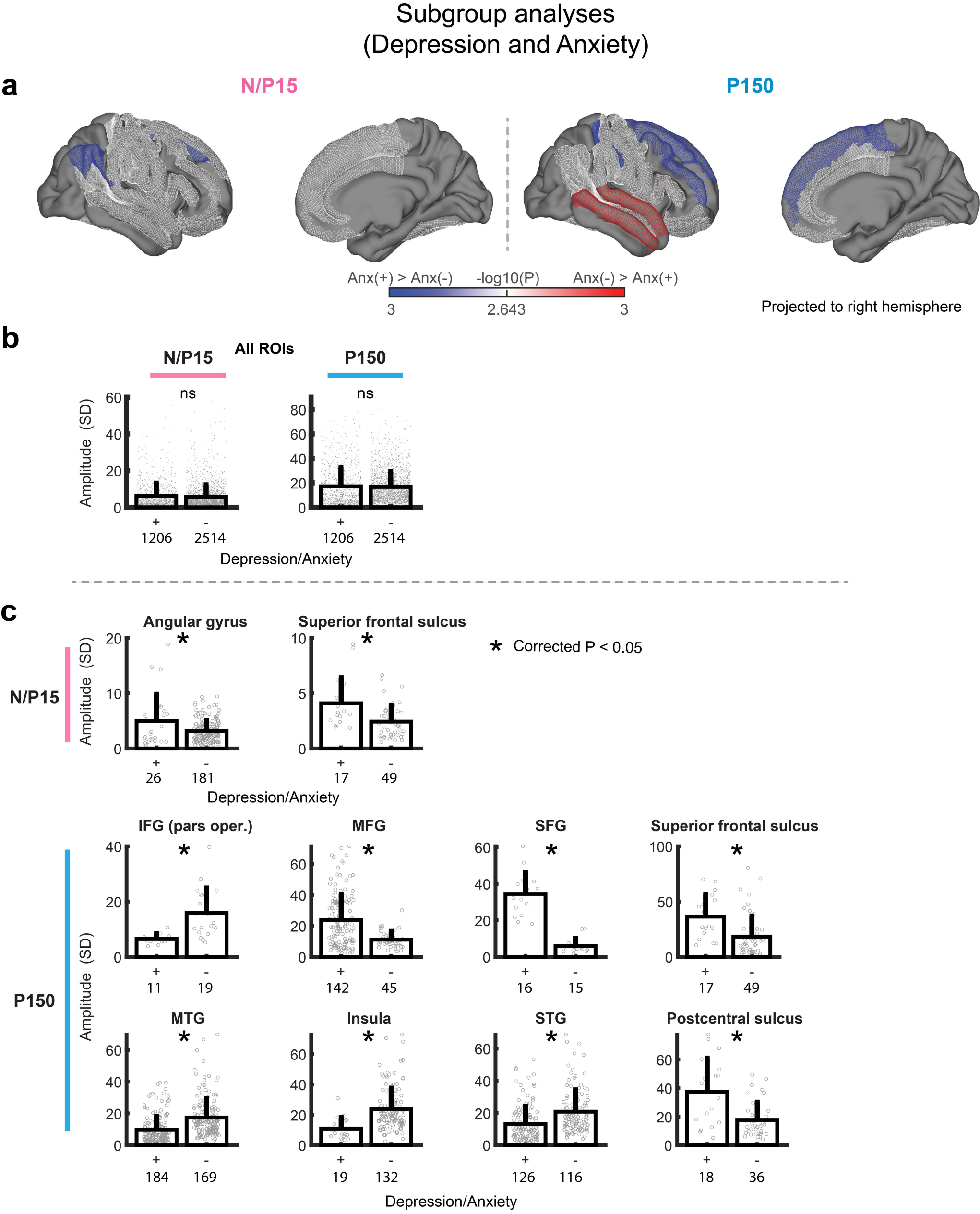

Supplementary Figure 9 | Amygdala stimulation es-TT response in patients with depression and/or anxiety. (a) ROIs that showed significant difference between patients with and without either depression or anxiety disorder (N = 5 and 8, respectively) are color-coded and shown on the template brain surface. ROIs in white mesh indicated non-significant ROIs. **(b)** es-TT response averaged over all ROIs for N/P15 (left) and P150 (right). Mean and SE are shown together with each data points represented by gray dots. Number of valid contacts are also shown below x axis. ns = non significant (two-sided t-tests, P>0.05). **(c)** Same as (b), but ROIs that showed significant difference (P < 0.05, Bonferroni correction, two-sided t-tests). Each data points are represented by gray circles. Here, medial and lateral group amygdala stimulations are combined. P values: N/P15: P = 6.74×10^{-4} and 9.83×10^{-3} for angular gyrus and superior frontal gyrus, respectively. P150: P = 2.19×10^{-3}, 1.39×10^{-6}, 9.18×10^{-10}, 1×10^{-3}, 5.83×10^{-12}, 8.77×10^{-5}, 2.22×10^{-6} and 1.7×10^{-4} for IFG, MFG, SFG, superior frontal sulcus, MTG, Insula, STG and postcentral sulcus, respectively. For the creation of brain backgrounds in panel (a), we used Conte69.32k surface mesh obtained from https://biomedia.doc.ic.ac.uk/brain-parcellationsurvey/) under WU-Minn HCP Consortium Open Access Data Use Terms.

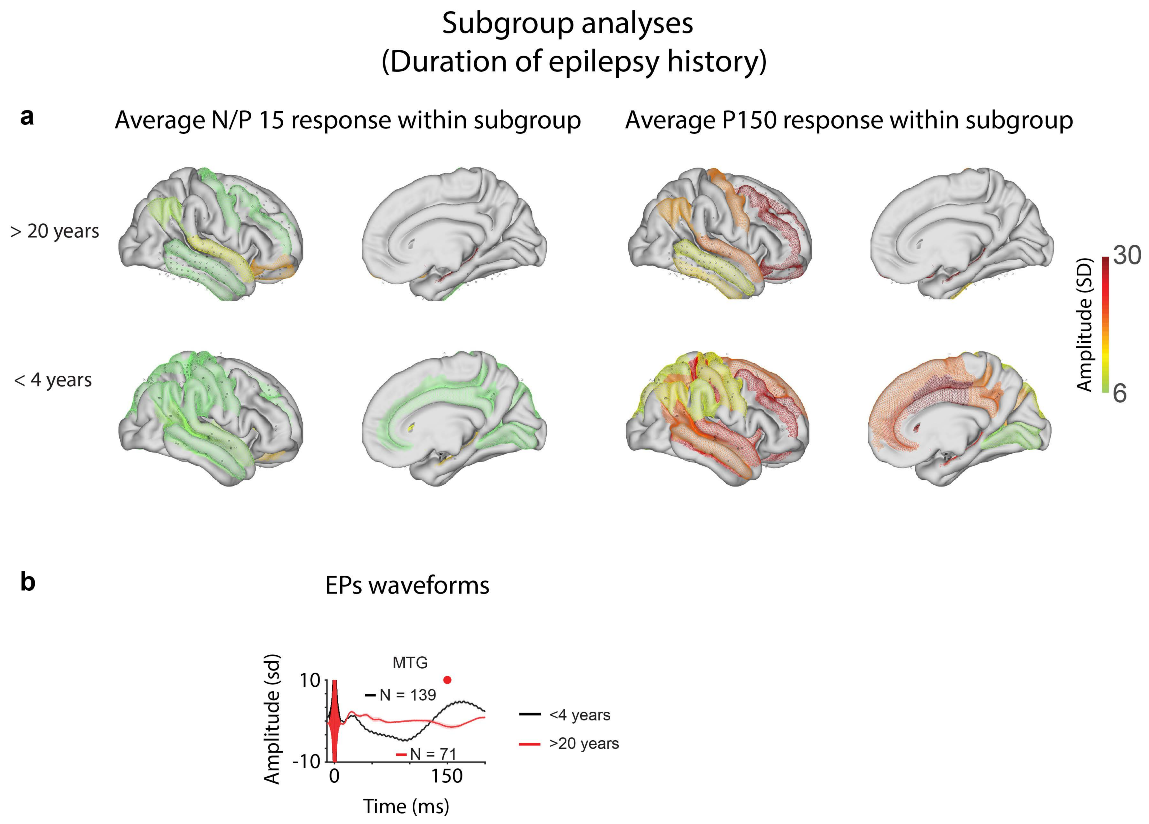

Supplementary Figure 10 | ROI averaged intracranial EPs waveform grouped by duration of epilepsy. Grouping was done using duration of epilepsy. **(a)** Group averaged es-TT response magnitudes mapped on to MNI brain. **(b)** ROI EPs that showed significant difference between groups. Red line and area: Overall averaged waveform and its SE (Epilepsy duration < 4 yr). Black line and area: Overall averaged waveform and its SE (Epilepsy duration > 20 yr). Red dots on the waveform indicate significantly different N/P15 (dots at 15 ms) or P150 component (dots at 150 ms) of EPs between two conditions ($P = 1.32 \times 10^{-6}$, two-sided t-test, Bonferroni correction). Only shows ROIs with number of contacts > 20 for each condition. Number of valid contacts within the ROI are also provided (N). Medial and lateral group stimulations were combined for the analyses. For the creation of brain backgrounds in panel (a), we used Conte69.32k surface mesh (obtained from https://biomedia.doc.ic.ac.uk/brain-parcellationsurvey/) under WU-Minn HCP Consortium Open Access Data Use Terms.

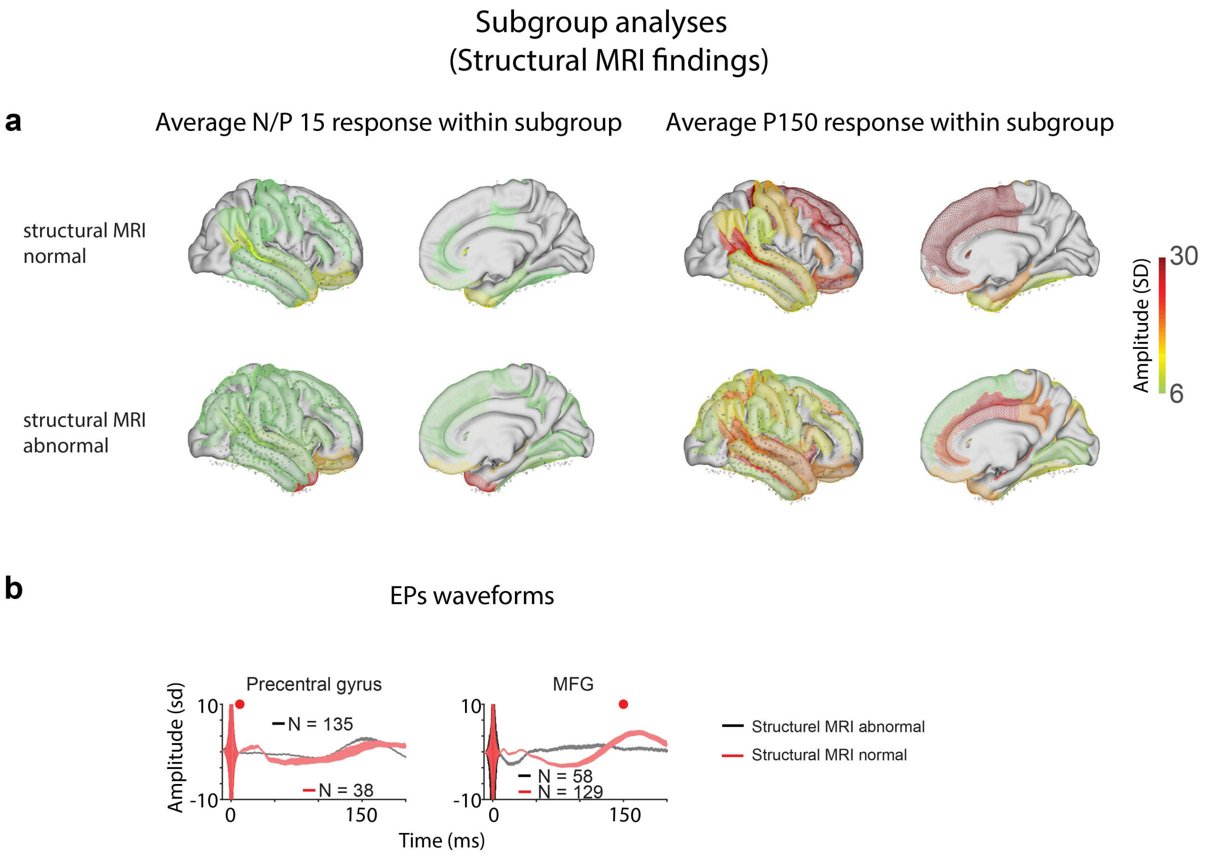

Supplementary Figure 11 | ROI averaged intracranial EPs waveform grouped by structural MRI findings. (a) Group averaged es-TT response magnitudes mapped on to MNI brain. **(b)** ROI averaged EPs that showed significant difference between groups. Red line and area: Overall averaged waveform and its SE (Structural MRI normal group). Black line and area: Overall averaged waveform and its se (Structural MRI abnormal group). Red dots on the waveform indicate significantly different N/P15 (dots at 15 ms) or P150 component (dots at 150 ms) of EPs between two conditions ($P = 8.81 \times 10^{-5}$ and 8.50×10^{-10} for precentral gyrus and MFG, respectively, Bonferroni correction, two-sided t-tests). Number of valid contacts within the ROI are also provided. Only ROIs that showed significant difference and had over 20 valid contacts are shown. Medial and lateral group stimulations were combined for the analyses. For the creation of brain backgrounds in panel (a), we used Conte69.32k surface mesh (obtained from https://biomedia.doc.ic.ac.uk/brain-parcellationsurvey/) under WU-Minn HCP Consortium Open Access Data Use Terms.

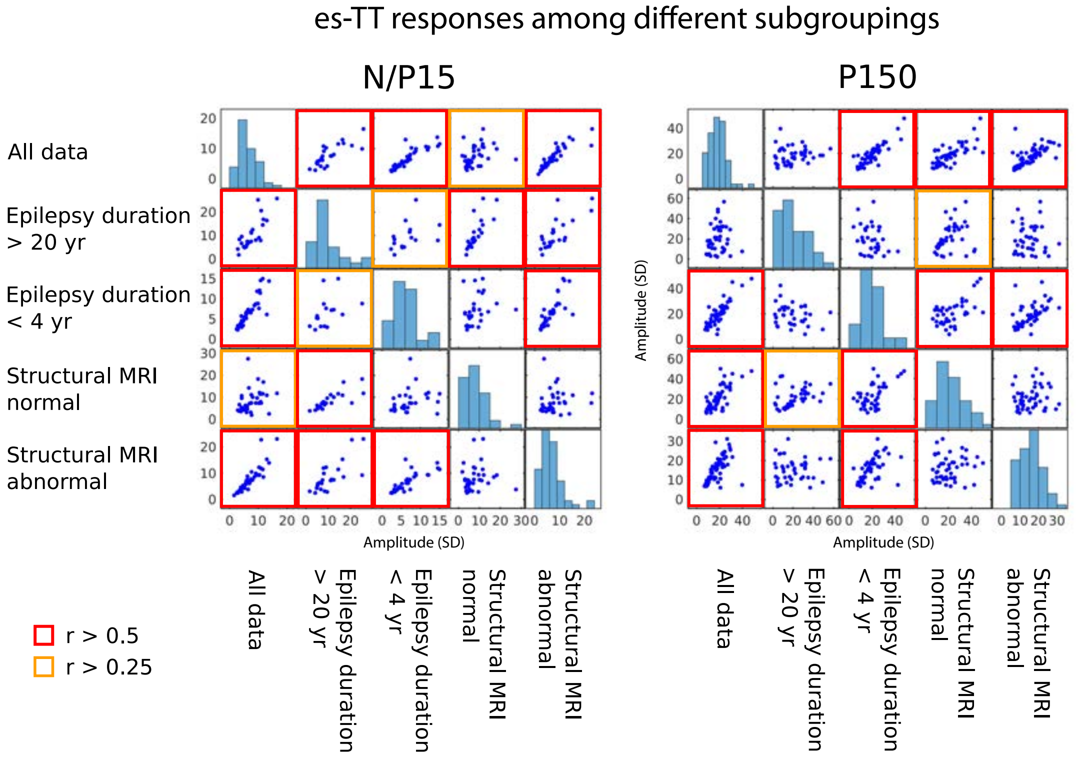

Supplementary Figure 12 | Comparisons across different subgroupings of patients. Each dot represents the es-TT response magnitude averaged within each structural ROI. Pearson correlation values (r) larger than 0.25 were indicated by colored (red or orange) axis-box. Medial and lateral group stimulations were combined for the analyses.

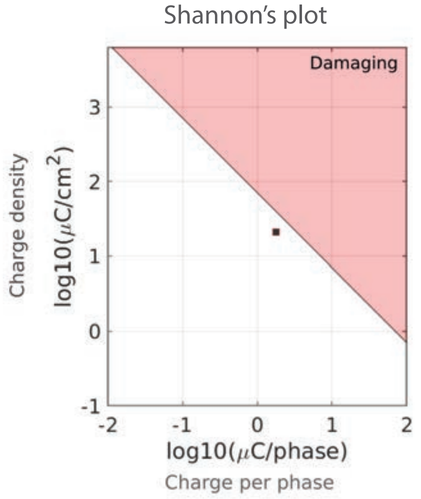

Supplementary Figure 13 | es-TT stimulation intensity. Stimulation intensity for es-TT is represented in charge density - charge per phase plane (Shannon's plot).

ID	esTT	es-fMRI	Stimulated hemisphere	Stimulated amygdala group es-TT	Stimulated amygdala group es-fMRI	Age	Sex	Handed-ness	Number of es-TT sites	Anxiety or depression	Duration of Epilepsy (y)	Clinical structural imaging finding	Surgical treatment
369	o	o	Left	Lateral	Both	30	Male	Right	2	(-)	4	Right basal ganglia venous anomaly	Right amygdalo-hippocampectomy
384	o	o	Right	Both	Medial	37	Male	Right	3	(+)	29	Normal	Right amygdalo-hippocampectomy
399	o	o	Right	Lateral	Lateral	21	Female	Right	2	(+)	12	Normal	Right amygdalo-hippocampectomy + right ventral frontal gliosis resection
405	o	o	Both	Both	Medial	19	Male	Right	5	(-)	10	Left frontal encephalomalacia	Electrode removal only
376	o		Right	Lateral		47	Female	Right	1	(+)	>20	Leukoaraiosis	Right amygdalo-hippocampectomy
400	o		Left	Lateral		59	Female	Right	2	(-)	7	Left mesial temporal sclerosis	Left amygdalo-hippocampectomy
404	o		Left	Both		43	Female	Right	2	(+)	29	Developmental malformation of left mesial temporal lobe	Left parietal cortical dysplasia resection
418	o		Right	Lateral		25	Female	Right	1	(-)	10	Right lateral temporal lobe cortical dysplasia	Right amygdalo-hippocampectomy
466	o		Left	Both		5	Male	Left	6	(-)	2	Venticulomegaly	Corpus callosotomy
511	o		Right	Medial		4	Female	unknown	4	(-)	4	Right parietal white matter T1 hyper-intensity	Right parietal glioneuronal lesion resection
515	o		Both	Both		21	Female	Right	5	(-)	3	Right hippocampal volume loss with	Right amygdalo-hippocampectomy
534	o		Right	Both		19	Male	Right	2	(-)	17	Status post left amygdalohippocampectomy, left temporal lobectomy	Bilateral anterior thalamus DBS
572	o		Left	Lateral		11	Male	Right	2	(+)	4	Normal	Electrode removal only
294		o	Right		Lateral	34	Male	Right		(-)	27	Normal	Rt frontal focus resection
303		o	Right		Medial	34	Female	Left		(+)	>20	Left fronto-temporal-parietal encephalomalasia	Electrode removal only
307		o	Left		Both	29	Male	Right		(-)	12	Cavernoma in posterior aspects of the left insula/temporal lobe	Left posterior insula cavernoma resection
322		o	Right		Medial	27	Male	Right		(-)	25	Cortical dysplasia within the right frontal lobe	Right frontal cortical dysplasia resection
330		o	Right		Medial	43	Male	Right		(-)	33	Focal cortical dysplasia in right occipital lobe.	Right lateral occipital cortex and right inferior temporal cortex resection
331		o	Right		Lateral	35	Male	Right		(+)	>20	Right medial temporal sclerosis	Electrode removal only
334		o	Both		Both	39	Male	Left		(-)	32	Right medial temporal lobe cystic lesion	Right temporal lobe ganglioglioma and focal cortical dysplasia resection
335		o	Left		Medial	31	Male	Right		(-)	24	Bilateral hippocampal abnormalities	Electrode removal only
352		o	Right		Medial	31	Male	Right/Left		(+)	19	Left frontal cystic lesion	Left frontal (IFG) cortical gliosis resection
372		o	Left		Lateral	33	Male	Right		(-)	2	Normal	Left amygdalo-hippocampectomy
395		o	Left		Both	13	Male	Right		(-)	4	Left frontal lobe cavernoma	Left frontal cavernoma resection
407		o	Right		Medial	13	Male	Right		(-)	7	Left frontal encephalomalacia	Electrode removal only
					mean and SD	28.1 + 13.3			2.85 + 1.63				

Supplementary Table 1 | Subject information

N/P 15

Anatomical ROI	Medial stimulation Mean ± SD	Lateral stimulation Mean ± SD	P (uncorrected)
Postcentral sulcus	3.35 ± 2.41 (N = 33)	8.93 ± 4.60 (N = 21)	3.40×10^{-7}
SPL	2.26 ± 1.54 (N = 87)	4.73 ± 1.12 (N = 12)	5.65×10^{-7}
ACC	3.29 ± 1.72 (N = 49)	7.28 ± 4.16 (N = 15)	1.01×10^{-6}
Transverse occipital sulcus	1.98 ± 1.06 (N = 42)	4.84 ± 1.81 (N = 6)	1.06×10^{-6}
Precentral gyrus	2.30 ± 1.75 (N = 120)	4.22 ± 3.21 (N = 53)	1.06×10^{-6}
Central sulcus	2.76 ± 1.55 (N = 39)	5.99 ± 3.19 (N = 22)	1.63×10^{-6}
Superior temporal sulcus	6.01 ± 4.50 (N = 111)	11.43 ± 10.41 (N = 76)	2.44×10^{-6}
Intraparietal sulcus	3.33 ± 1.93 (N = 20)	6.85 ± 0.80 (N = 10)	7.38×10^{-6}
Postcentral gyrus	2.50 ± 1.32 (N = 64)	4.22 ± 2.79 (N = 60)	1.96×10^{-5}
Subparietal sulcus	3.79 ± 2.38 (N = 150)	6.64 ± 3.47 (N = 13)	1.03×10^{-4}
Orbital gyri	19.53 ± 13.65 (N = 29)	9.55 ± 10.94 (N = 61)	3.40×10^{-4}
Short insula gyri	5.81 ± 5.81 (N = 34)	12.87 ± 4.24 (N = 10)	9.38×10^{-4}

P150

Anatomical ROI	Medial stimulation Mean ± SD	Lateral stimulation Mean ± SD	P (uncorrected)
MTG	14.34 ± 10.00 (N = 156)	7.33 ± 5.61 (N = 197)	1.96×10^{-15}
MFG	22.79 ± 10.12 (N = 60)	11.60 ± 8.73 (N = 127)	5.43×10^{-13}
STG	17.96 ± 10.80 (N = 93)	9.33 ± 7.03 (N = 149)	1.01×10^{-12}
Orbital gyri	24.30 ± 11.40 (N = 29)	9.06 ± 6.13 (N = 61)	1.40×10^{-12}
Transverse occipital sulcus	5.73 ± 3.71 (N = 42)	14.83 ± 2.45 (N = 6)	5.65×10^{-7}
IFG (pars triang.)	23.35 ± 7.37 (N = 7)	9.53 ± 5.28 (N = 30)	1.49×10^{-6}
Transverse frontopolar	34.43 ± 16.90 (N = 3)	7.01 ± 2.90 (N = 13)	2.41×10^{-5}
ITG	15.33 ± 14.47 (N = 20)	6.79 ± 6.22 (N = 83)	9.10×10^{-5}
Inferior temporal sulcus	22.76 ± 13.74 (N = 46)	10.33 ± 3.41 (N = 17)	5.07×10^{-4}

Supplementary Table 2 | Over all es-TT responses (mean and SD) in which significant difference between medial and lateral group stimulation were observed. Two-sided t-tests (Bonferroni correction). Uncorrected P values were reported.

Anatomical site	peak T	size	Peak x	Peak y	Peak z
Overall response					
Bilateral posterior cingulate gyrus	-9	3018 *	0.5	28.5	31.5
bilateral thalamus	-5.99	3018 *	5.5	14.5	2.5
left precuneus	-5.45	1040 **	5.5	74.5	36.5
Right precuneus	-5.13	1040 **	13.5	74.5	43.5
Right insula	7.4	1327	-41.5	-1.5	3.5
Bilateral anterior cingulate gyrus	5.4	342	0.5	-23.5	-6.5
Right fusiform gyrus	-5.67	327	-49.5	48.5	-46.5
Left insula	4.37	160	42.5	-5.5	-4.5
Left middle cingulate gyrus	4.42	159	4.5	-3.5	33.5
Left superior frontal gyrus	5.19	138	18.5	-9.5	63.5
left middle temporal gyrus	5.63	125	66.5	22.5	-24.5
Left orbitofrontal gyrus	5.51	124	14.5	-25.5	-16.5
right precentral gyrus	5.08	93	-49.5	2.5	43.5
Left angular gyrus	-4.87	89	44.5	62.5	39.5
Right pre- and post- central gyrus	5.07	78	-46.5	13.5	47.5
right superior temporal gyrus	5.26	72	-52.5	10.5	-2.5

*,** combined

Medial – vs – Lateral contrast					
bilateral thalamus	-5.29	299	-3.5	26.5	3.5
right precentral gyrus	6.58	202	-17.5	10.5	77.5
Left precentral gyrus	-4.65	174	45.5	22.5	58.5
left middle temporal gyrus	-6.4	169	62.5	0.5	-20.5
Bilateral superior frontal gyrus (medial)	-5.4	163	2.5	-61.5	7.5
left precuneus	-5.2	124	2.5	72.5	35.5
Right inferior frontal gyrus	4.81	116	-51.5	-23.5	21.5
Right angular gyrus	5.49	100	-57.5	46.5	51.5
right superior temporal gyrus	5.42	70	-67.5	32.5	2..5
Right middle frontal gyrus*	-4.75	58	-43.5	-31.5	43.5
Right posterior cingulate gyrus*	-4.17	52	8.5	50.5	35.5
Left precentral gyrus*	5.49	45	14.5	22.5	71.5

* cluster size or 45
was used for these clusters

Supplementary Table 3 | Results of es-fMRI group analysis. Upper table shows significant clusters for overall response (medial and lateral group stimulations were combined). Lower table shows contrast between medial and lateral group stimulation.
