# Supplementary material for: Mapping effective connectivity of human amygdala subdivisions with intracranial stimulation
Source: Nat Commun. 2022 Aug 20;13:4909. doi: 10.1038/s41467-022-32644-y (PMC9392722; doi:10.1038/s41467-022-32644-y)
Supplement: Supplementary file 3 — Reporting Summary [file 41467_2022_32644_MOESM3_ESM.pdf]

nature portfolio

Corresponding author(s): Hiroyuki Oya

Last updated by author(s): Jun 28, 2022

Reporting Summary

Nature Portfolio wishes to improve the reproducibility of the work that we publish. This form provides structure for consistency and transparency in reporting. For further information on Nature Portfolio policies, see our Editorial Policies and the Editorial Policy Checklist.

Statistics

For all statistical analyses, confirm that the following items are present in the figure legend, table legend, main text, or Methods section.

n/a	Confirmed	
☐	☒	The exact sample size (*n*) for each experimental group/condition, given as a discrete number and unit of measurement
☐	☒	A statement on whether measurements were taken from distinct samples or whether the same sample was measured repeatedly
☐	☒	The statistical test(s) used AND whether they are one- or two-sided<br>*Only common tests should be described solely by name; describe more complex techniques in the Methods section.*
☐	☒	A description of all covariates tested
☐	☒	A description of any assumptions or corrections, such as tests of normality and adjustment for multiple comparisons
☐	☒	A full description of the statistical parameters including central tendency (e.g. means) or other basic estimates (e.g. regression coefficient) AND variation (e.g. standard deviation) or associated estimates of uncertainty (e.g. confidence intervals)
☐	☒	For null hypothesis testing, the test statistic (e.g. F, t, r) with confidence intervals, effect sizes, degrees of freedom and P value noted<br>*Give P values as exact values whenever suitable.*
☒	☐	For Bayesian analysis, information on the choice of priors and Markov chain Monte Carlo settings
☒	☐	For hierarchical and complex designs, identification of the appropriate level for tests and full reporting of outcomes
☐	☒	Estimates of effect sizes (e.g. Cohen's *d*, Pearson's *r*), indicating how they were calculated

Our web collection on statistics for biologists contains articles on many of the points above.

Software and code

Policy information about availability of computer code

Data collection	iEEG data collection: Neuralynx Pegasus v2.22<br>es-fMRI data collection: Siemens NUMARIS, Syngo MR Ell
Data analysis	MR Image analysis: FreeSurfer (v. 7.2.0)<br>iEEG/es-fMRI data analysis: Matlab 2019b (Mathworks)<br>iEEG analysis: R (v. 4.0.2) Catboost:<br>R package catboost (v. 0.23.2) UMAP:<br>R package umap (v. 0.2.7.0)<br>es-fMRI data analysis: AFNI (v. 20.3.01)<br>Community detection: R package leidenAlg (v. 1.0.2)<br>R package igraph (1.3.2)

For manuscripts utilizing custom algorithms or software that are central to the research but not yet described in published literature, software must be made available to editors and reviewers. We strongly encourage code deposition in a community repository (e.g. GitHub). See the Nature Portfolio guidelines for submitting code & software for further information.

Data

Policy information about availability of data

All manuscripts must include a data availability statement. This statement should provide the following information, where applicable:

- Accession codes, unique identifiers, or web links for publicly available datasets
- A description of any restrictions on data availability
- For clinical datasets or third party data, please ensure that the statement adheres to our policy

es-fMRI data is available at OpenNeuro database. Identifier: DOI 10.17605/OSF.IO/DNGV5
es-TT data is available in Open Science Framework (OSF) database. OpenNeuro Accession Number: ds002799

Human research participants

Policy information about studies involving human research participants and Sex and Gender in Research.

Reporting on sex and gender	Our analysis includes biological sex as an factor and there was no sex - gender differences in the participants recruited in this study.
Population characteristics	Patients with intractable epilepsy (adult and pediatric) were recruited and participated in the study. There was no exclusion due to race, gender, duration of epilepsy, structural MRI findings and other psychiatric conditions. See Table SI.
Recruitment	Patients with intractable epilepsy who required invasive monitoring of their epilepsy were recruited. Decision for the invasive monitoring was determined by The University of Iowa multidisciplinary clinical epilepsy treatment team in which physicians not involved in the research were also the member. This process occurred before the recruitment to the research study and free from any selection bias for research. As a result, our participants demographics were diverse (Table S1) and sub-grouping within our participants gave similar results.
Ethics oversight	The university of Iowa institutional review board and California Institute of Technology Institutional Review Board approved all the experimental protocol used in this study.<br>If the participant was minor (<18 y/o), parents gave written informed consent.

Note that full information on the approval of the study protocol must also be provided in the manuscript.

Field-specific reporting

Please select the one below that is the best fit for your research. If you are not sure, read the appropriate sections before making your selection.

☒ Life sciences ☐ Behavioural & social sciences ☐ Ecological, evolutionary & environmental sciences

For a reference copy of the document with all sections, see nature.com/documents/nr-reporting-summary-flat.pdf

Life sciences study design

All studies must disclose on these points even when the disclosure is negative.

Sample size	25 neurosurgical patients with epilepsy who required invasive monitoring of their epilepsy. See materials and methods. Sample size in this study was not pre-determined by power analysis, but rather determined by the patient/subject availability during the study periods.
Data exclusions	Recordings that contained non-physiological artifacts were excluded.
Replication	We did not consider separate replication group due to availability of the subjects (epilepsy patients). However, sub-grouping (splitting) within our patients gave similar results. We also performed cross-validation in our machine learning analyses in both es-TT and es-fMRI data to show the stable differentiation between the conditions over all participants. The experiments were usually conducted once per stimulation site per patients.
Randomization	No randomization was done. This study is not a clinical trial. Our subjects are all epilepsy patients who needed invasive monitoring. The experiments need implantation of intracranial electrodes which is not possible for normal control or other patients. All participants underwent the same protocol.
Blinding	Not applicable. All subjects in this study were patients with epilepsy who were indicated for invasive iEEG monitoring by clinical team.

Reporting for specific materials, systems and methods

We require information from authors about some types of materials, experimental systems and methods used in many studies. Here, indicate whether each material, system or method listed is relevant to your study. If you are not sure if a list item applies to your research, read the appropriate section before selecting a response.

Materials & experimental systems

n/a	Involved in the study
☒	☐ Antibodies
☒	☐ Eukaryotic cell lines
☒	☐ Palaeontology and archaeology
☒	☐ Animals and other organisms
☒	☐ Clinical data
☒	☐ Dual use research of concern

Methods

n/a	Involved in the study
☒	☐ ChIP-seq
☒	☐ Flow cytometry
☐	☒ MRI-based neuroimaging

Magnetic resonance imaging

Experimental design

Design type: Block design

Design specifications: 10 blocks of electrical stimulation (es) ON and OFF. 220 frames. 30 sec es-ON and 30 sec es-OFF. Total duration for one es-fMRI run was 11 min. Multiple es-fMRI runs were possible in one session in some patients.

Behavioral performance measures: The data was acquired in resting condition. No active performance measure was collected.

Acquisition

Imaging type(s): Functional MRI (GE-EPI)

Field strength: 3.0 Tesla

Sequence & imaging parameters: 2D gradient-echo echo planar imaging sequence. TR = 3.0s, TE = 30ms, Slice thickness = 3.0 mm, FOV = 220 mm, Flip Angle = 90 degrees. Pixel bandwidth = 1935 Hz/Pixel. 68 x 68 matrix. Phase encoding direction = Anterior-Posterior

Area of acquisition: whole brain

Diffusion MRI ☐ Used ☒ Not used

Preprocessing

Preprocessing software: fMRIPrep 1.2.3

Normalization: Yes, unwarped (with field map) functional imaging datasets were spatially normalized to MNI template with non-linear morphing using a combination of affine transformation and antsRegistration (available in ANTS).

Normalization template: ICBM152NLinAsym

Noise and artifact removal: Noise removal was done using regression. 6 motion parameters, their temporal derivatives and top 6 aCompcor components were used as noise regressors (total of 18 noise regressors). Spatial smoothing was also applied using gaussian kernel of 6.0 mm FWHM width.

Volume censoring: Yes. The EPI volume that exceeded framewise displacement (FD) larger than 0.9 mm and one frame before that is censored.

Statistical modeling & inference

Model type and settings: Mass-univariate GLMs (general linear models with prewhitened time series) with variable shape regression (using cubic spline function expansion) were used for the first-level analyses. Second-level analyses were done with mixed-effect analysis with 3dMEMA (AFNI).

Effect(s) tested: Effect of electrical stimulation (es) was assessed first with mass-univariate GLM for each es-fMRI run. Next, difference of es effect between medial and lateral group of the amygdala stimulation was examined using mixed-effect analysis and multivariate classification.

Specify type of analysis: ☒ Whole brain ☐ ROI-based ☐ Both

Statistic type for inference (See Eklund et al. 2016): Cluster-wise alpha = 0.02 with primary voxel-wise threshold P = 0.01 was used.

Correction	Simulation-based cluster size threshold was determined with 3dClustSim (AFNI). Spatial autocorrelation was fit to gaussian plus exponential model.

Models & analysis

n/a	Involved in the study
☐	☒ Functional and/or effective connectivity
☐	☒ Graph analysis
☐	☒ Multivariate modeling or predictive analysis

Functional and/or effective connectivity	Residual BOLD time series resulted from deconvolution were averaged for each ROI, and this was used to calculate connectivity matrices. Full partial correlations were used.
Graph analysis	Weighted connectivity matrices resulted from the partial correlation analyses were parcellated with Leiden community detection algorithm.
Multivariate modeling and predictive analysis	ROI averaged BOLD activations across whole brain were used for stimulus site classification (medial versus lateral group amygdala stimulation) using RandomForest classification.
